# The mitochondrial genetic landscape in neuroblastoma from tumor initiation to relapse

**DOI:** 10.18632/oncotarget.6776

**Published:** 2015-12-28

**Authors:** Lara M. Riehl, Johannes H. Schulte, Medhanie A. Mulaw, Meike Dahlhaus, Matthias Fischer, Alexander Schramm, Angelika Eggert, Klaus-Michael Debatin, Christian Beltinger

**Affiliations:** ^1^ Department of Pediatrics and Adolescent Medicine, University Medical Center Ulm, Ulm, Germany; ^2^ Pediatric Oncology and Hematology, Charité University Medicine, Berlin, Germany; ^3^ German Cancer Research Center (DKFZ), German Cancer Consortium (DKTK), Heidelberg, Germany; ^4^ Institute for Experimental Cancer Research, University Medical Center Ulm, Ulm, Germany; ^5^ Core Facility Genomics, Faculty of Medicine, Ulm University, Ulm, Germany; ^6^ Department of Pediatric Oncology and Hematology, University Children's Hospital Cologne, Medical Faculty, University of Cologne, Cologne, Germany; ^7^ Center for Molecular Medicine Cologne (CMMC), University of Cologne, Cologne, Germany; ^8^ Department of Pediatric Oncology and Hematology, University Children's Hospital Essen, Essen, Germany

**Keywords:** mitochondrial variants, neuroblastoma, tumor progression, next generation sequencing, phylogenetic analysis

## Abstract

Little is known about changes within the mitochondrial (mt) genome during tumor progression in general and during initiation and progression of neuroblastoma (NB) in particular. Whole exome sequencing of corresponding healthy tissue, primary tumor and relapsed tumor from 16 patients with NB revealed that most NB harbor tumor-specific mitochondrial variants. In relapsed tumors, the status of mt variants changed in parallel to the status of nuclear variants, as shown by increased number and spatio-temporal differences of tumor-specific variants, and by a concomitant decrease of germline variants. As mt variants are present in most NB patients, change during relapse and have a higher copy number compared to nuclear variants, they represent a promising new source of biomarkers for monitoring and phylogenetic analysis of NB.

## INTRODUCTION

The mt genome differs from the nuclear genome by its high copy number, small size, intronless genomic structure, different mode of replication, exposure to oxygen radicals and increased DNA damage [1, 2]. While it is well-established that tumor-specific mt variants and mutations are present in many cancers, little is known about alterations of the mt genome during tumor relapse [3].

NB is the most common extracranial solid tumor of childhood, with a poor prognosis in advanced stages [4]. The nuclear genome of NB patients carries only a small number of mutations [5–7]. At relapse, the nuclear genome of NB frequently harbors mutations in the RAS-MAPK pathway [8] and shows an increased mutational burden, a shift in the mutational spectrum, reduced mutational heterogeneity and continuing evolution [9]. It is unknown whether NB harbor alterations in their mt genome, whether these change during relapse and how this change compares with that of nuclear variants. We show here that the majority of NB patients have tumors with evolving mt variants, providing the foundation for novel means to analyze and monitor NB.

## RESULTS

**Figure 1:**
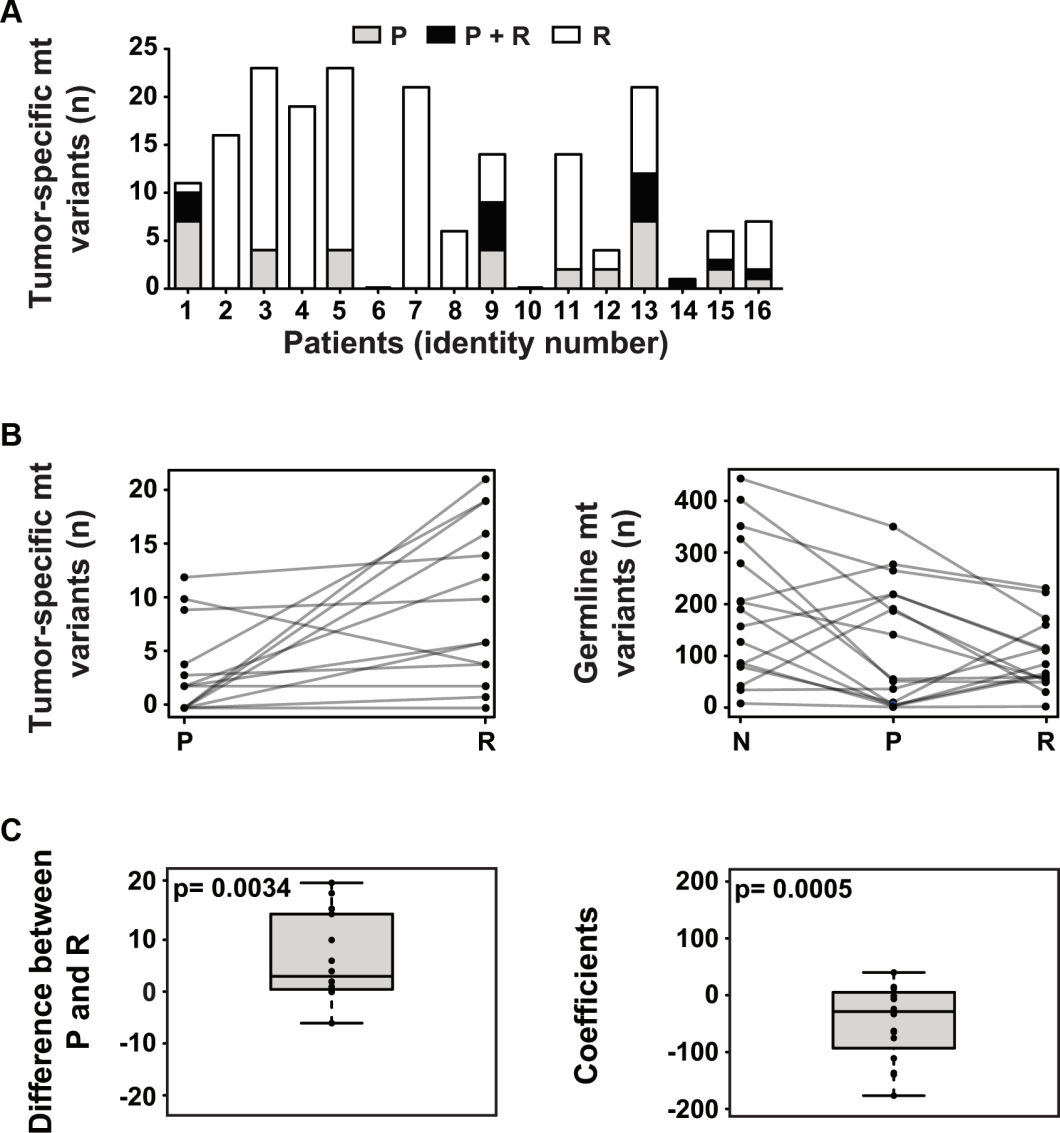
The mitochondrial genome in NB (**A**) The majority of NB harbors tumor-specific mt variants, with increased frequency at relapse. The number of tumor-specific mt variants per patient present in the primary tumor at diagnosis (P), relapsed tumor (R) or both (P + R) is shown. (**B**) The number of tumor-specific mt variants increases at relapse while the number of germline mt variants decreases during tumor initiation and relapse. In the left panel, the number of tumor-specific mt variants per patient in the primary (P) and the relapsed (R) tumor is shown. In the right panel, the number of germline mt variants per patient in normal tissue (N), primary tumor (P) and relapsed tumor (R) is depicted. (**C**) At relapse, the number of tumor-specific and germline mt variants significantly increases and decreases, respectively. In the left panel, the paired differences of tumor-specific variants between primary (P) and relapsed tumor (R) of each patient are plotted. The null hypothesis of the mean of the paired differences being equal to 0 was tested by Monte Carlo simulation and the exact *p*-value was calculated. For the right panel, a linear regression model was fitted for each patient. The slope coefficients of the fitted lines were used to depict the trend. The distribution of the coefficients is plotted. The null hypothesis, i.e. the mean of these coefficients being 0 when the number of variants in normal tissue, primary tumor and relapse are randomly distributed, was tested by Monte Carlo simulation and the exact *p*-value was calculated.

To check whether mt DNA alterations are present in NB, we analyzed mt sequences extracted from whole exome sequencing data of corresponding normal tissue, primary (diagnostic) tumors and relapsed tumors of 16 NB patients (Supplementary Table 1) that we previously had investigated for nuclear mutations [9]. 14 of 16 patients (87.5%) had tumors with tumor-specific mt variants. Mt variants ranged from 0 to 21 variants per tumor (mean 6.3), 65.0% of which occurred in relapsed disease (Figure 1A and Table 1). All of these variants were heteroplasmic and most of them had a low allele frequency (Supplementary Table 2). In total, 19 previously unpublished tumor-specific variants (Supplementary Table 2) and 28 recurrent tumor-specific variants (Supplementary Table 3) were found, most of them silent. All tumors possessed germline mt variants ranging from 1 to 350 per tumor (mean 112.3) (Table 1).

**Table 1:** Number of tumor-specific and germline mt variants with corresponding differences and coefficients

Patient	Tumor-specific mt variants (*n*)^a^		Difference^b^	Germline mt variants (*n*)^c^		Coefficient^d^
	P	R		P	R	
1	10	4	−6	187	51	−175.5
2	0	16	16	5	66	−62.0
3	4	19	15	191	30	−6.0
4	0	19	19	10	160	40.5
5	4	19	15	265	223	−64.0
6	0	0	0	219	110	−23.5
7	0	21	21	3	84	−1.0
8	0	6	6	36	115	40.5
9	9	10	1	277	231	12.5
10	0	0	0	55	59	−110.0
11	2	12	10	3	62	−32.5
12	2	2	0	51	49	−138.5
13	12	14	2	219	113	15.0
14	0	1	1	1	2	−3.0
15	3	4	1	350	172	−135.5
16	2	6	4	141	55	−74.5

a Number of tumor-specific mt variants: P, primary tumor; R, relapse tumor. Variants with an allele frequency of at least 1% were analyzed.

b Difference: Pairwise difference between primary and relapse tumor.

c Number of germline mt variants: P, primary tumor; R, relapse tumor. Variants with an allele frequency of at least 1% were analyzed.

d Coefficient: Coefficient as calculated by linear regression.

Next, we wanted to know whether the status of mt variants changes during the course of NB. Indeed, in 14 out of 16 patients, changes from tumor at diagnosis to relapse were seen (Supplementary Table 2). In particular, an increase in the number of tumor-specific mt variants occurred at relapse in 12 of these patients (Figure 1B, left panel) leading to a positive mean difference of variants proved to be statistically significant (Figure 1C, left panel). Concomitantly, changes of germline mt variants at relapse were seen in all patients (Table 1). In particular, a decrease of germline mt variants from normal tissue to relapse in 10 out of 16 patients (Figure 1B, right panel) caused a statistically significant negative mean difference of germline variants (Figure 1C, right panel). Most NB showed a concordant increase of tumor-specific variants in the nuclear and mt genomes from primary to relapsed tumor (Supplementary Figure 1). None of the recurrently mutated genes of the nuclear genome was associated with an increased number of tumor-specific mt variants (data not shown).

**Figure 2:**
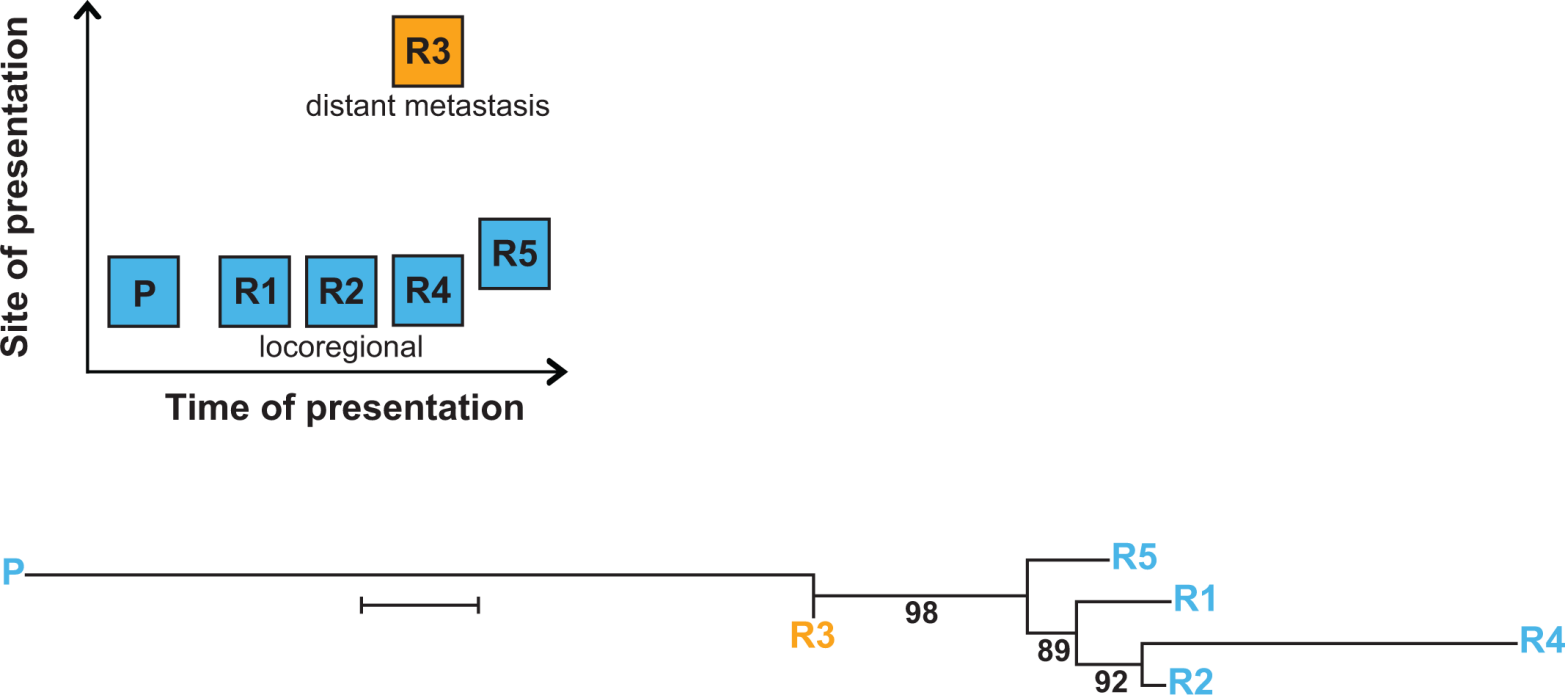
Multiple relapses in patient #12 reveal spatio-temporal change of tumor-specific mt variants The upper panel shows time and site of occurrence of the primary tumor and the relapses. The lower panel depicts the branching of relapse samples R1–R5 from the primary tumor P. Branch lengths reflect the phylogenetic distance between tumor samples (bar equals 0.2 base substitutions per site), numbers represent bootstrap values (%) supporting a given branching point. Phylogenetic reconstruction was performed using the Neighbor-Joining method for evolutionary history and the Jukes-Cantor method for evolutionary distances.

An unique opportunity to investigate the phylogeny of NB relapse arose in patient #12, who suffered a series of five relapses, the third (R3) and forth (R4) of which occurred concurrently at different sites (Figure 2, upper panel). In this patient, tumor-specific mt variants not only occurred late, i.e. at third relapse, but were also site-dependent, as R3 and R4 harbored mutually exclusive mt variants (Supplementary Table 2). A phylogenetic reconstruction analysis of the relapsed tumor samples utilizing their mt variants revealed two independent branches originating from the primary tumor. One branch subdivided to the locoregional relapses R1, R2, R4 and R5, and the other branch to R3, a distant metastasis (Figure 2, lower panel). While R1, R2 and R4 were strictly local, R5 occurred more distant in a regional lymph node.

**Figure 3:**
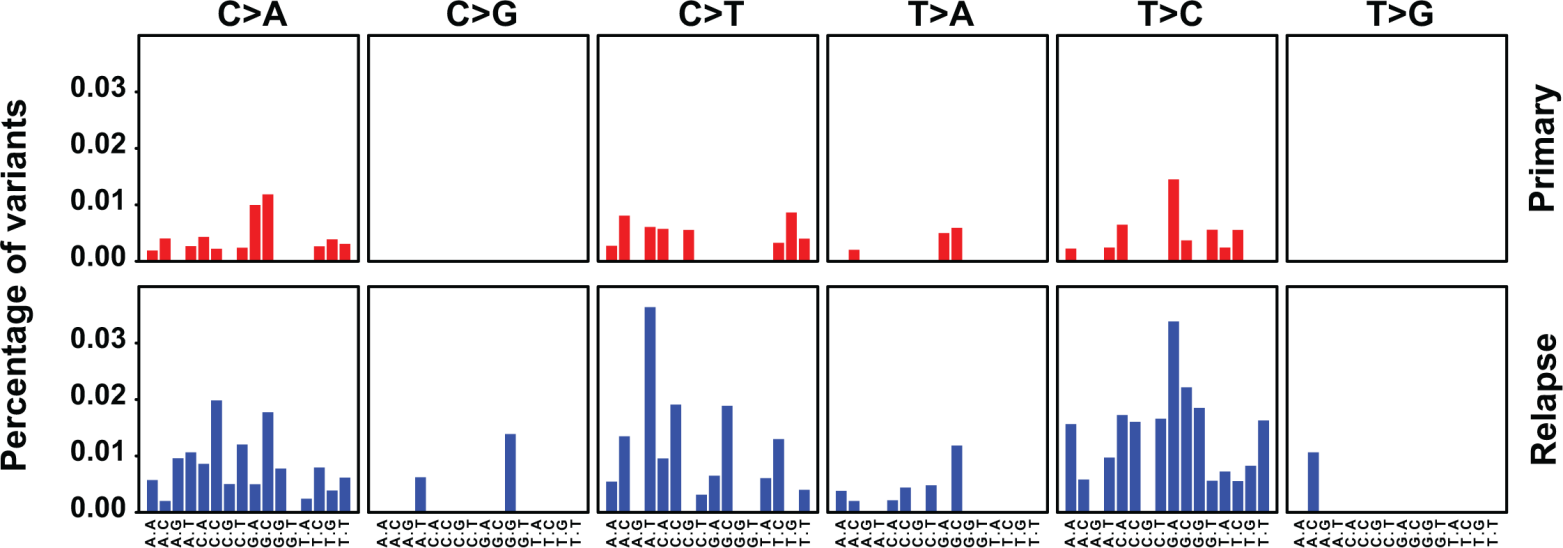
The mutation spectrum of the mt genome suggests replication-dependent DNA damage and does not change at relapse Primary and relapsed tumors are compared. The 6 base substitutions classes are indicated on the top and the bases flanking the substitutions 5′ and 3′ are depicted at the bottom. The percentages show the observed frequencies of the base substitutions within their sequence context normalized by the frequency seen in the Revised Cambridge Reference Sequence (rCRS) of the mt genome.

Finally, we assessed how the mutational spectrum of the mt genome evolves during progression of NB in patients. To this end, the mutational signature of primary and relapsed tumors were obtained comparing mt variant motif spectra during tumor initiation and relapse. The spectrum of mutations at initial disease was consistent with replication-induced but not with reactive oxygen species-induced DNA damage [3] and did not change at relapse (Figure 3).

## DISCUSSION

In this study we show for the first time that the majority of NB harbors mt DNA variants and that relapsing NB displays enhanced evolutional changes. The changes of the mt variants in NB at relapse, as manifested by increased number and spatio-temporal differences of tumor-specific variants, and by the concomitant decrease of germline variants, most likely has been caused by heterogeneous genetic drift of mt variants, given that selection of mt variants has only been observed with nonsense mutations [3]. Cumulated heterogeneity of mt variants may lead to differences in the activity of oxidative phosphorylation and mitochondrial apoptosis [1]. These time- and site-dependent changes may dictate rebiopsy of relapses and metastases, if the latest state of mt variants is to be determined comprehensively. Whether intratumoral heterogeneity of mt changes also exists, requires future investigations.

We previously investigated the same set of corresponding primary and relapsed NB for the dynamics of nuclear mutations [9]. Despite the many structural and functional differences of the mt and nuclear genomes, their variants showed parallels during progression. Thus, at relapse both genomes concomitantly harbored increased tumor-specific recurrent and non-recurrent variants, and had decreased germline variants. Both genomes also showed spatio-temporal heterogeneity during progression of NB. An association of specific recurrent nuclear mutations with an increase of tumor-specific mt variants was not evident in this small cohort.

While mitochondrial phylogenetic analysis of relapses in patient #12 was congruent with the corresponding nuclear analysis [9] in delineating two major branches of relapses, it diverged in the assignment of R1 and R5. This divergence may be attributed to differences between the mt and the nuclear genome in copy number, size and replication, leading to different mechanisms of heterogeneity. These data show that mitochondrial phylogenetic analysis yields information complementary to nuclear phylogenetics.

Mutation spectra differed between the mt and nuclear genomes, suggesting different mutational mechanisms. This may be explained by the differences in genomic structure, replication, DNA damage and DNA repair between the two genomes. The stable mt mutation spectrum of primary and relapsed tumors suggests that similar mutational mechanisms act upon the mt genome at the time of diagnosis and relapse. In contrast, the changing nuclear mutation spectrum [9] proposes that mutating mechanisms in the nuclear genome change during tumor progression.

Taken together, we have determined the presence and change of mt DNA variants during neuroblastomagenesis. Notwithstanding whether mt variants have functional consequences and represent actionable targets in NB, they are a promising source of biomarkers for monitoring and phylogenetic analysis of NB.

## MATERIALS AND METHODS

### Patients

Corresponding blood and tumor tissue at diagnosis and at relapse were collected from 16 NB patients diagnosed and treated in participating study centers of the German neuroblastoma trials NB 97 and 2004. All participants provided informed consent. Detailed patient characteristics can be found in Supplementary Table 1.

### Exome capture, library preparation and massively parallel sequencing

These methods are described in detail elsewhere [9]. Briefly, exome capture and library preparation were performed using the NimbleGen SeqCap EZ Human Exome Library SR kits v2 or v3 (Roche, Basel, Switzerland) and the TruSeq Sample Preparation kit v2 (Illumina, San Diego, CA, USA).

### Mitochondrial data filtering

Whole exome sequencing data was filtered for mitochondrial sequences and screened for mt variants using the MitoSeek tool [10] and the revised Cambridge Reference Sequence. Thresholds were set to a coverage of > 50 reads per base. The mean coverage of mitochondrial variants was 212. An abundance of at least 2% was chosen as a threshold for tumor-specific variants and germline variants and at least 1% for investigating changes of variants. Resulting variants were compared and evaluated using MITOMAP: A Human Mitochondrial Genome Database. (http://www.mitomap.org) and mtDB: Human Mitochondrial Genome Database (http://www.mtdb.igp.uu.se/).

### Dynamics of mitochondrial variants

**Tumor-specific mt variants.** Corresponding primary and relapse tumor samples were first compared to normal tissue to identify tumor-specific mt variants. The paired difference between primary tumor and relapse for every patient was taken to assess the change in number of tumor-specific mt variants across all patient samples. To obtain a single parameter expressing the changes of all mt variants, the mean of the paired differences between primary tumor and relapse was calculated. The null hypothesis of the mean of the paired differences being equal to 0 when there is no increment or decrement of variants across sample types was tested. To this end, Monte Carlo simulation with 10,000 iterations was performed using randomly generated uniform numbers within the range of the observed values. The exact *p*-value was calculated.

**Germline mt variants.** A linear regression model was fitted for every patient and the slope of the fitted line was taken as the coefficient that describes the trend of changes of the number of variants between normal tissue, primary tumor and relapse. To obtain a single parameter expressing the changes of all mt variants across all patients and samples, the mean of these coefficients was calculated. The null hypothesis, i.e. the mean of these coefficients being 0 when the number of variants in normal tissue, primary tumor and relapse are randomly distributed, was tested by Monte Carlo simulation and the exact *p*-value was calculated.

### Mitochondrial phylogenetic analysis

All mt variants that passed the frequency filtering threshold (1%; 346 variants from the primary tumor sample and its 5 relapses) were used for the reconstruction of the phylogenetic tree in MEGA6 [11]. The evolutionary history was inferred using the Neighbor-Joining method [12]. The evolutionary distances were computed using the Jukes-Cantor method [13]. The unit is the number of base substitutions per site. Gamma distribution (shape parameter = 0.75) was used to model the rate variation among sites. The confidence probability (multiplied by 100) of the interior branch length being greater than 0 was estimated using the bootstrap test (1,000 replicates) [14, 15]. The tree was drawn to scale with the branch lengths reflecting the evolutionary distances.

### Mutation spectrum analysis

We adapted a method previously described [3, 16] to obtain a mutational signature of the mt variants specific for primary and relapse tumor samples. Briefly, we first calculated the frequencies of the 6 classes of base changes within their 5′ and 3′ flanking bases (96 possible combinations) for the primary and relapse tumor samples separately. The frequencies of these changes were also calculated for the Revised Cambridge Reference Sequence (rCRS) of the mt genome and used to normalize the frequencies of the changes in the primary and relapse samples. Results were then displayed as bar plots with the y-axis representing the normalized frequencies and the x-axis showing the base changes and their 5′ and 3′ flanking bases.

### General statistical analysis

The GraphPad Prism 6.01 software (La Jolla, CA, USA) was used.

## SUPPLEMENTARY MATERIALS FIGURE AND TABLES


