# Supplementary material for: The mitochondrial genetic landscape in neuroblastoma from tumor initiation to relapse
Source: Oncotarget. 2015 Dec 28;7(6):6620–5. doi: 10.18632/oncotarget.6776 (PMC4872737; doi:10.18632/oncotarget.6776)
Supplement: Supplementary file 1 [file oncotarget-07-6620-s001.pdf]

The mitochondrial genetic landscape in neuroblastoma from tumor initiation to relapse

Supplementary Materials

A

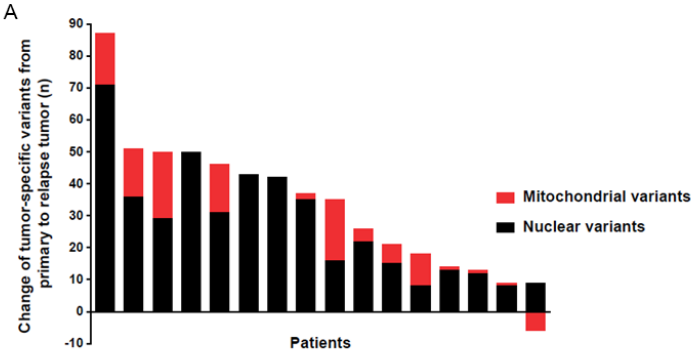

B

	Patients with increase in variants	Patients without increase in variants	Marginal row totals
Mitochondrial variants	12	4	16
Nuclear variants	16	0	16
Marginal column totals	28	4	32

Supplementary Figure S1: Concomitant increase of tumor-specific variants in the nuclear and mt genomes from primary to relapsed NB. (**A**) Increase in tumor-specific variants in both the nuclear and the mt genomes of paired primary and relapsed NB at relapse. Shown are the number of tumor-specific variants in the nuclear (black) and mt (red) genome that changed in the relapsed tumor compared to the primary tumor of each patient. Spearman‘s rank correlation test resulted in a *r*-value of 0.0607 and a *p*-value of 0.8232. (**B**) Concordant increment of tumor-specific variants in both genomes at relapse. Concordance between the nuclear and mt changes in tumor-specific variants from primary tumors to relapse tumors was evaluated using Fisher‘s exact test, resulting in a *p*-value of 0.1012. Patients without increase include patients with decreasing and unchanging variants.

Supplementary Table S1: Patient characteristics

Patient	Gender	Stage[a]	Age at diagnosis[b]	Tumor[c]	*MYCN*[d]
1	f	4	514	P	a
				R1	a
2	m	4	989	P	a
				R2	a
3	m	2b	810	P	a
				R1	a
4	f	4	1964	P	a
				R1	a
5	m	4	1371	P	a
				R1	a
6	f	4s	119	P	na
				R1	a
7	m	4	416	P	a
				R1	na
8	f	4	1221	P	na
				R1	na
9	m	4	2337	P	na
				R1	na
10	f	2b	1787	P	na
				R3	na
11	m	4	1812	P	na
				R1	na
12	f	2a	4273	P	na
				R1	na
				R2	na
				R3	nd
				R4	na
				R5	na
13	m	3	1408	P	na
				R1	na
14	f	4	1520	P	nd
				R1	nd
15	m	4	1444	P	na
				R1	na
16	m	2a	59	P	na
				R1	h

[a]International Neuroblastoma Staging System.
[b]Age at diagnosis in days.
[c]P, primary tumor; R1-R5: first, second, third, fourth and fifth relapse.
[d]a, amplified; na, not amplified; nd, not determined; h, heterogeneous.

Supplementary Table S2: Tumor-specific mt variants

Patient	Variant position and substitution[a]						Variant allele frequency (%)[b]					
							P	R1	R2	R3	R4	R5
1	16sRNA	2259	C	>	T	nc	4.4	2.9				
	COX1	7337	G	>	A	Ser478Ser	< 1	3.2				
	ND4	10778	C	>	A	*Pro7Thr	2.1	< 1				
	ND4	11719	A	>	G	Gly320Gly	2.1	< 1				
	ND5	13326	T	>	C	Cys330Cys	2.9	< 1				
	ND5	13680	C	>	T	Pro448Pro	4.8	3.5				
	tRNAGlu	14727	T	>	C	nc	2.9	< 1				
	CYTB	14766	T	>	C	Ile7Thr	6.1	< 1				
	CYTB	14872	C	>	T	Ile42Ile	4.6	2.0				
	CYTB	15773	G	>	A	Val343Met	5.9	< 1				
	D-Loop	16519	C	>	T	nc	3.5	< 1				
2	D-Loop	73	A	>	G	nc	< 1		2.5			
	D-Loop	252	T	>	C	nc	< 1		2.6			
	D-Loop	477	C	>	T	nc	< 1		3.6			
	ND2	5249	T	>	C	Phe266Phe	< 1		2.5			
	COX1	6146	A	>	G	Trp81Trp	< 1		2.4			
	COX1	7028	C	>	T	Ala375Ala	< 1		2.2			
	COX3	9380	A	>	G	Trp58Trp	< 1		7.1			
	ND4	10907	T	>	C	Phe50Leu	< 1		2.0			
	ND4	11009	T	>	C	Leu84Leu	< 1		2.8			
3	12sRNA	1243	T	>	C	nc	< 1	3.0				
	16sRNA	2159	T	>	C	nc	4.3	< 1				
	16sRNA	2301	T	>	C	nc	< 1	3.5				
	16sRNA	2706	A	>	G	nc	< 1	3.1				
	ND1	3505	A	>	G	Thr67Ala	< 1	3.6				
	COX1	6528	C	>	T	Leu209Leu	< 1	2.6				
	COX1	7028	C	>	T	Ala357Ala	< 1	3.0				
	COX2	7830	G	>	A	Arg82His	5.4	< 1				
	ATP6	8994	G	>	A	Leu156Leu	< 1	4.1				
	ND3	10097	A	>	G	Leu13Leu	< 1	2.4				
	ND4	11674	C	>	T	Thr305Thr	< 1	3.3				
	ND4	11719	G	>	A	Gly320Gly	< 1	4.5				
	ND4	11947	A	>	G	Thr396Thr	< 1	2.5				
	ND5	12414	T	>	C	Pro26Pro	< 1	2.2				
	ND5	12705	C	>	T	Ile123Ile	< 1	2.4				
	ND6	14193	A	>	G	Phe161Leu	5.2	< 1				
	CYTB	14766	C	>	T	Ile7Thr	< 1	3.0				
	CYTB	15326	G	>	A	Thr194Ala	< 1	3.0				
	CYTB	15775	A	>	G	Val343Val	< 1	2.9				
	D-Loop	16223	C	>	T	nc	< 1	2.7				
	D-Loop	16292	C	>	T	nc	< 1	2.9				
	D-Loop	16305	A	>	T	nc	< 1	2.6				
	D-Loop	16311	T	>	C	nc	5.7	< 1				

4	D-Loop	152	C	>	T	nc	< 1	4.1				
	D-Loop	195	C	>	T	nc	< 1	7.1				
	D-Loop	499	A	>	G	nc	< 1	4.5				
	16sRNA	2289	G	>	A	*nc	< 1	3.4				
	16sRNA	3010	G	>	A	nc	< 1	3.2				
	COX1	7028	T	>	C	Ala357Ala	< 1	4.1				
	COX2	7645	T	>	C	Leu20Leu	< 1	3.7				
	ATP6	8818	T	>	C	Leu98Leu	< 1	7.8				
	ND4	11719	A	>	G	Gly320Gly	< 1	3.3				
	tRNAHis	12172	A	>	G	nc	< 1	2.9				
	ND5	14034	T	>	C	Ile566Ile	< 1	4.0				
	ND6	14484	T	>	C	Met64Val	< 1	6.2				
	ND6	14620	T	>	C	Gly18Gly	< 1	8.0				
	CYTB	14766	T	>	C	Ile7Thr	< 1	4.3				
	CYTB	15544	A	>	C	Pro266pro	< 1	4.8				
	CYTB	15693	C	>	T	Thr316Met	< 1	5.8				
	D-Loop	16256	C	>	T	nc	< 1	2.7				
	D-Loop	16311	T	>	C	nc	< 1	2.1				
	D-Loop	16356	C	>	T	nc	< 1	8.0				
5	D-Loop	73	A	>	G	nc	< 1	6.5				
	D-Loop	152	T	>	C	nc	< 1	5.9				
	D-Loop	217	T	>	C	nc	< 1	3.0				
	16sRNA	2718	C	>	A	*nc	< 1	2.0				
	ND1	3992	T	>	C	Met229Thr	< 1	6.2				
	ND2	5390	A	>	G	Met307Met	< 1	2.0				
	COX1	6480	G	>	A	Val193Ile	< 1	5.2				
	COX2	7768	A	>	G	Met61Met	2.2	< 1				
	ATP6	9055	G	>	A	Ala177Thr	< 1	2.4				
	ND4	11197	C	>	T	Gly146Gly	< 1	2.6				
	ND4	11467	A	>	G	Leu236Leu	< 1	2.7				
	ND4	11719	G	>	A	Gly320Gly	< 1	7.7				
	ND4	11732	T	>	C	Leu325Leu	< 1	5.9				
	tRNALeu2	12308	A	>	G	nc	3.7	< 1				
	ND5	12372	G	>	A	Leu12Leu	< 1	3.7				
	ND5	13708	G	>	A	Ala458Thr	5.5	< 1				
	ND6	14446	C	>	A	*Glu76Asp	2.3	< 1				
	CYTB	14766	C	>	T	Thr7Ile	< 1	6.5				
	CYTB	14798	T	>	C	Phe18Leu	< 1	2.0				
	CYTB	15693	T	>	C	Met316Thr	< 1	2.8				
	D-Loop	16179	C	>	T	nc	< 1	4.0				
	D-Loop	16209	T	>	C	nc	< 1	2.3				
	D-Loop	16263	C	>	T	nc	< 1	14.7				
	D-Loop	16304	T	>	C	nc	< 1	3.8				
	D-Loop	16519	C	>	T	nc	< 1	2.5				
7	16sRNA	1811	G	>	A	nc	< 1	3.8				
	16sRNA	2706	G	>	A	nc	< 1	2.3				
	16sRNA	3010	G	>	A	nc	< 1	2.4				

	ND2	4811	G	>	A	Trp114Trp	< 1	3.3				
	ND2	4968	T	>	C	*Trp167Arg	< 1	5.1				
	ND2	5342	T	>	C	Tyr291Tyr	< 1	4.8				
	COX1	5985	A	>	G	Ile28Val	< 1	3.6				
	COX1	5999	C	>	T	Ala32Ala	< 1	3.9				
	COX1	6047	G	>	A	Leu48Leu	< 1	3.7				
	COX1	6146	G	>	A	Trp81Trp	< 1	2.6				
	COX1	7028	T	>	C	Ala375Ala	< 1	4.0				
	ATP6	9070	G	>	T	Ala128Ser	< 1	2.9				
	ND4	11467	G	>	A	Leu236Leu	< 1	4.0				
	ND4	11719	A	>	G	Gly320Gly	< 1	3.3				
	ND6	14620	T	>	C	Gly18Gly	< 1	2.0				
	CYTB	14766	T	>	C	Ile7Thr	< 1	3.4				
	CYTB	14866	T	>	C	Cys40Cys	< 1	4.6				
	D-Loop	16126	T	>	C	nc	< 1	2.2				
	D-Loop	16145	G	>	A	nc	< 1	2.2				
	D-Loop	16172	T	>	C	nc	< 1	2.0				
	D-Loop	16356	C	>	T	nc	< 1	7.8				
	D-Loop	16519	C	>	T	nc	< 1	2.6				
8	D-Loop	340	C	>	A	nc	< 1	3.3				
	tRNAHis	12172	A	>	G	nc	< 1	2.9				
	ND5	12937	A	>	G	Met201Val	< 1	2.1				
	CYTB	15043	G	>	A	Gly99Gly	< 1	2.1				
	CYTB	15693	T	>	C	Met316Thr	< 1	2.5				
	D-Loop	16519	T	>	C	nc	< 1	2.7				
9	D-Loop	95	A	>	C	nc	< 1	3.4				
	D-Loop	152	T	>	C	nc	4.3	5.6				
	D-Loop	499	G	>	A	nc	5.1	3.0				
	COX1	6047	A	>	G	Leu48Leu	< 1	2.5				
	COX1	6182	G	>	A	Ala93Ala	3.5	4.3				
	COX2	7645	T	>	C	Leu20Leu	< 1	8.5				
	COX3	9947	A	>	G	Val247Val	9.2	< 1				
	ND5	12937	A	>	G	Met201Val	2.1	2.2				
	ND5	13404	T	>	C	Ile356Ile	2.9	4.3				
	ND6	14620	C	>	T	Gly18Gly	3.0	< 1				
	CYTB	15544	C	>	A	Pro266Pro	< 1	3.8				
	CYTB	15758	A	>	G	Ile338Val	5.2	< 1				
	D-Loop	16134	C	>	T	nc	3.1	< 1				
	D-Loop	16352	T	>	C	nc	< 1	3.6				
11	HV3	456	T	>	C	nc	< 1	3.0				
	16sRNA	709	A	>	G	nc	< 1	3.2				
	16sRNA	1811	A	>	G	nc	< 1	2.0				
	16sRNA	1880	C	>	A	*nc	< 1	2.2				
	16sRNA	2487	A	>	C	*nc	< 1	2.0				
	16sRNA	2570	C	>	T	*nc	5.1	< 1				
	16sRNA	3010	G	>	A	nc	< 1	3.3				
	COX2	7028	C	>	T	Ala375Ala	< 1	2.2				

	ATP6	8084	G	>	A	Ala167Thr	< 1	2.5				
	ATP6	9053	A	>	G	Arg176Ser	< 1	4.6				
	ND4	9070	T	>	G	Ser128Ala	< 1	2.5				
	ND4	11009	T	>	C	Leu84Leu	< 1	2.3				
	CYTB	11169	G	>	A	*Gly137Asp	2.1	< 1				
	CYTB	14766	C	>	T	nc	< 1	2.3				
12	12sRNA	1338	A	>	G	*nc	< 1	< 1	21.8	< 1	27.6	7.9
	ND2	5053	C	>	A	*Pro195Glu	< 1	< 1	< 1	< 1	2.1	< 1
	ATP6	8781	C	>	G	*Leu85Leu	4.1	< 1	< 1	< 1	< 1	< 1
	COX3	9840	T	>	C	Ser212Pro	< 1	< 1	< 1	< 1	41.1	31.2
	ND3	10306	A	>	G	*Arg83Ser	< 1	< 1	3.9	< 1	< 1	< 1
	ND4L	10531	T	>	C	*Val21Ala	3.5	< 1	< 1	< 1	< 1	< 1
	ND4	11827	T	>	C	Ala356Ala	< 1	< 1	< 1	< 1	2.0	< 1
	ND4	12129	T	>	C	Phe457Ser	< 1	< 1	< 1	< 1	8.2	< 1
	ND5	12367	A	>	G	Thr11Ala	< 1	< 1	< 1	7.0	< 1	< 1
13	D-Loop	73	A	>	G	nc	4.9	2.7				
	D-Loop	152	T	>	C	nc	4.9	3.1				
	12sRNA	1360	G	>	A	*nc	2.0	< 1				
	16sRNA	3010	A	>	G	nc	2.6	5.0				
	COX1	5999	T	>	C	Ala32Ala	2.5	< 1				
	COX1	6047	A	>	G	Leu48Leu	2.0	< 1				
	COX1	6385	C	>	A	*Ala161Asp	2.0	< 1				
	ATP6/8	8572	A	>	G	Gly16Ser/ Stop-69Stop	3.8	< 1				
	ND4	11332	C	>	T	Ala191Ala	2.1	< 1				
	tRNAHis	12172	G	>	A	nc	3.4	5.0				
	ND5	12501	G	>	A	Met55Met	3.8	< 1				
	ND5	12705	C	>	T	Ile123Ile	5.4	< 1				
	ND5	12937	A	>	G	Met201Val	2.3	< 1				
	ND5	13404	T	>	C	Ile356Ile	3.7	< 1				
	ND5	13416	A	>	G	Gly360Gly	2.1	2.1				
	ND6	14438	G	>	A	*Pro79Leu	< 1	2.0				
	ND6	14620	C	>	T	Gly18Gly	2.7	< 1				
	CYTB	15043	G	>	A	Gly99Gly	< 1	3.2				
	CYTB	15693	T	>	C	Met316Thr	< 1	2.1				
	CYTB	15758	A	>	G	Ile338Val	5.6	< 1				
	D-Loop	16337	C	>	A	nc	2.1	< 1				
	D-Loop	16356	T	>	C	nc	< 1	2.1				
	D-Loop	16051	A	>	G	nc	< 1	4.7				
	D-Loop	16362	T	>	C	nc	< 1	4.1				
	D-Loop	16390	G	>	A	nc	< 1	2.1				
14	ND5	13153	A	>	G	Ile273Val	< 1	12.9				
15	16sRNA	3010	G	>	A	nc	< 1	2.2				
	COX1	6272	A	>	G	Gly123Gly	3.6	< 1				
	ND4L	10695	G	>	A	Ala76Thr	15.6	< 1				
	ND5	13131	C	>	A	*Pro265Pro	< 1	2.0				
	CYTB	14869	G	>	A	Leu41Leu	3.9	2.2				

	tRNAThr	15904	T	>	C	nc	< 1	5.3				
16	ND1	3492	A	>	C	Lys62Asn	3.5	< 1				
	COX1	6419	A	>	C	Lys172Asn	5.2	5.1				
	COX1	6968	C	>	A	*Gly355Gly	2.3	< 1				
	ND5	13368	G	>	A	Gly344Gly	< 1	2.9				
	CYTB	15607	A	>	G	Lys287Lys	< 1	2.5				
	D-Loop	16163	A	>	G	nc	< 1	5.0				
	D-Loop	16294	C	>	T	nc	< 1	3.3				

[a]Variant position and substitution: Gene or region in the mitochondrial genome, variant position in the mitochondrial genome, base and amino acid substitution. Previously unpublished variants according to MITOMAP are marked with an asterisk (*). ATP6/8, ATP synthase subunit 6 and 8; COX1, COX2 and COX3 cytochrome c oxidase subunit 1, 2 and 3; CYTB, cytochrome b; D-Loop, displacement loop; HV3, hypervariable region 3; nc, non coding; ND1-6, mt encoded NADH dehydrogenase 1-6; tRNAGlu, tRNAHis, tRNALeu2 and tRNAThr, tRNA Glutamin, Histidine, Leucine 2 and Threonine; 12sRNA and 16sRNA, 12s and 16s ribosomal RNA.

[b]Percentage of mitochondrial allele frequency. P, primary tumor; R1–R5: first to fifth relapse; < 1, allele frequency below the threshold set at 1%.

Supplementary Table S3: Recurrent tumor-specific mt variants

Variant position and substitution[a]						Tumors with variants (*n*)		Mean variant allele frequency[b] (%)	
						**P**	**R**	**P**	**R**
D-Loop	73	A	>	G	nc	1	3	4.9	3.9
D-Loop	152	C	>	T	nc	2	4	4.3	5.2
D-Loop	499	A	>	G	nc	1	2	5.1	3.8
16sRNA	1811	A	>	G	nc	0	2	< 1	2.9
16sRNA	2706	A	>	G	nc	0	2	< 1	2.7
16sRNA	3010	G	>	A	nc	1	5	2.3	3.0
COX1	5999	C	>	T	Ala32Ala	1	1	2.5	3.9
COX1	6047	A	>	G	Leu48Leu	1	2	2.0	3.1
COX1	6146	A	>	G	Trp81Trp	0	2	< 1	2.5
COX1	7028	C	>	T	Ala375Ala	0	4	< 1	3.1
COX1	7645	T	>	C	Leu20Leu	0	2	< 1	6.1
ATP6	9070	G	>	T	Ala128Ser	0	2	< 1	2.7
ND4	11009	T	>	C	Leu84Leu	0	2	< 1	2.6
ND4	11467	A	>	G	Leu236Leu	0	2	< 1	3.4
ND4	11719	A	>	G	Gly320Gly	1	4	2.1	4.5
tRNAHis	12172	A	>	G	nc	1	3	3.4	4.0
ND5	12705	C	>	T	Ile123Ile	1	1	5.4	2.4
ND5	12937	A	>	G	Met201Val	2	2	2.2	2.2
ND5	13404	T	>	C	Ile356Ile	2	1	3.3	4.3
ND6	14620	T	>	C	Gly18Gly	2	2	2.9	5.0
CYTB	14766	C	>	T	Ile7Thr	1	5	6.1	3.9
CYTB	15043	G	>	A	Gly99Gly	0	2	< 1	2.7
CYTB	15544	A	>	C	Pro266Pro	0	2	< 1	4.3
CYTB	15693	C	>	T	Met316Thr	0	4	< 1	4.3
CYTB	15758	A	>	G	Ile338Val	2	0	5.4	< 1

D-Loop	16311	T	>	C	nc	1	1	5.7	2.1
D-Loop	16356	C	>	T	nc	0	3	< 1	7.9
D-Loop	16519	C	>	T	nc	0	4	< 1	3.5

[a]Variant position and substitution: Gene or region in the mitochondrial genome, variant position in the mitochondrial genome, base and amino acid substitution. COX1, cytochrome c oxidase subunit 1; CYTB, cytochrome b; D-Loop, displacement loop; nc, non coding; ND4-6, mt encoded NADH dehydrogenase 4–6; tRNAHis, tRNA Histidine; 16sRNA, 16s ribosomal RNA.

[b]Percentage of mitochondrial allele frequency. P, primary tumor; R, relapse tumor; < 1, allele frequency below the threshold set at 1%.
